# Landmine injuries at the Emergency Management Center in Erbil, Iraq

**DOI:** 10.1186/1752-1505-4-15

**Published:** 2010-08-18

**Authors:** Nazar P Shabila, Husen I Taha, Tariq S Al-Hadithi

**Affiliations:** 1 Department of Community Medicine, College of Medicine, Hawler Medical University, Erbil, Iraq; 2 Department of Surgery, College of Medicine, Hawler Medical University, Erbil, Iraq

## Abstract

**Background:**

Landmines can cause death, injury and disability in addition to many indirect public health consequences. This study aimed at understanding the trends, demography and other epidemiological characteristics of hospitalized landmine injured patients in Erbil governorate.

**Methods:**

The case records of landmine injured patients who had been admitted to the Emergency Management Centre in Erbil city from July 1998 to July 2007 were reviewed and descriptively analyzed.

**Results:**

Two hundred eighty five landmine injured patients were admitted to the center, their mean ± SD age was 26.5 ± 13.2 years (range 6-71 years), 95.1% were males, nearly 50% were between 19 to 35 years of age and 96.8% were civilians. Around 72% of victims sustained limb amputations; 58.6% lower limb and 13.3% upper limb out of the total. The hospital mortality rate was 2.1%. The number of admissions for landmine injury was steadily decreasing between July 1998 and July 2001, followed by prominent increase between July 2002 and July 2003. The highest proportion of admissions occurred in summer (35.4%) and majority of incidents occurred along the borders with Iran and Turkey (61.8%).

**Conclusion:**

Civilian male adolescents and young adults constituted the majority of hospitalized landmine victims in Erbil governorate. While a high proportion of victims sustained lower limb amputations, upper limb amputations particularly among children and injury to head and face were relatively common which might be attributed to handling explosives. This emphasizes the need to examine the reasons behind handling explosives.

## Background

Landmines have been used widely and indiscriminately throughout the world. They frequently result in devastating effects mainly among civilians in post conflict situations. In addition to causing death, injury and disability, landmines have many indirect public health consequences on civilian populations like being an important economic threat through preventing access to large areas of land and thus hindering agriculture work, livestock herding and infrastructure improvement [1-3]. They also cause displacement of population and are a frequent reason for preventing the return of internally displaced persons and refugees to their homes [4]. Landmines remain a risk for decades after being deployed and can entail substantial financial burden on individuals and communities [1].

The precise scale of the worldwide landmine problem is unknown as there is no systematic collection of reliable data on victims [2]. Available data on casualties are mainly limited to hospital-based data as only few countries possess community-based data collection system on landmine victims [5,6]. There are very few studies that have conducted surveillance and reported the amount of injuries and deaths due to landmines that occur in communities [7]. It is widely estimated that landmines result in 15,000-25,000 victims each year [5,6].

Most landmine accidents occur in developing countries including countries that have been overwhelmed by wars and have inadequate health and rehabilitation facilities [1]. Those that are hospitalized for landmine injury are mainly civilians, especially adult males, living in poor and remote rural areas [1,7-10]. A high proportion of victims fail to receive appropriate health care and there is usually a high pre-hospital mortality rate among them [11,12].

Iraq is severely affected by landmines as a result of the different wars and internal conflicts over the last four decades. Landmines are widely deployed throughout the country especially in the northern Kurdistan region along the Iraqi-Iranian border [5,13]. Research is scant and knowledge is limited about the public health consequences of the use of landmines in Iraq. Therefore, this study reviews 9 years data of landmine victims admitted to Emergency Management Centre (EMC) in Erbil governorate in order to understand the trends, demography and other epidemiological characteristics of hospitalized landmine injured patients. Such understanding may help in designing and guiding programs for prevention of landmine injuries in Erbil Governorate.

## Methods

Erbil governorate is located in northern Iraq and is inhabited by approximately two million persons, comprising 8 administrative districts that are distributed in both mountainous and plain geographical areas. Erbil governorate has borders with Iran and Turkey as well as the central part of Iraq, which was previously called the green zone. Erbil city is the capital of Iraqi Kurdistan region.

EMC is the only and definitive center to provide in-hospital care for all war wounded including landmine injuries in Erbil governorate, whether the victims are civilians or militants. It also occasionally receives patients from other governorates of Iraq. All injuries occurring outside the territories of Erbil governorate were excluded from the study. Minor injuries not requiring hospitalization are usually treated at district hospitals and do not reach EMC.

The case records of patients admitted to EMC in Erbil from July 13, 1998 through July 12, 2007 suffering from landmine injuries were reviewed using a standardized form for this purpose. These records included hand written admission and discharge summaries in addition to demographic characteristics of the victims. The starting date of the study is based on the date of establishment of EMC as a special center for treating war-wounded in Erbil governorate including landmine victims. Data on age and sex of patients, type of injury, location of incident and diurnal, monthly and yearly occurrence of injuries were extracted from the case records. The study excluded those injured by unexploded ordinance (UXO) other than landmines, as they were recorded in the log entries with other shell injuries resulting from terrorist and suicide incidents. Statistical analysis involves only application of descriptive statistics. This study was approved by the Ethics Committee at Hawler Medical University and by the EMC.

## Results

During the study period, 285 patients were admitted to EMC for landmine injuries. These injuries have resulted from 255 incidents including 27 multi-causality incidents; 24 incidents resulted in injury of 2 persons per incident and 3 incidents resulted in injury of 3 persons per incident. Out of 27 multi-casualty incidents, 5 incidents led to injury of first degree relatives with a total of 13 victims; 2 incidents led to injury of 2 relatives each and 3 incidents led to injury of 3 relatives each.

The mean ± SD age of the landmine victims was 26.5 ± 13.2 years (range 6-71 years). Those between 19 and 35 years of age constituted nearly 50% of victims and males constituted 95.1%. Details of age and sex distribution of victims are shown in Table 1. Civilians constituted 96.84% of victims. None of the victims was a landmine cleaner.

**Table 1:** Age and sex distribution of landmine victims

Age group (years)	Male		Female		Total	
	
	**No**.	(%)	**No**.	(%)	**No**.	(%)
**Children**						
0-6	1	(0.4)	0	(0.0)	1	(0.4)
7-12	26	(9.6)	0	(0.0)	26	(9.1)
13-18	61	(22.5)	5	(35.7)	66	(23.2)
**Total**	**88**	**(32.5)**	**5**	**(35.7)**	**93**	**(32.6)**

**Adults**						
19-35	134	(49.5)	7	(50.0)	141	(49.5)
≥ 35	49	(18.1)	2	(14.3)	51	(17.9)
**Total**	**183**	**(67.5)**	**9**	**(64.3)**	**192**	**(67.4)**

**Grand total**	**271**	**(100.0)**	**14**	**(100.0)**	**285**	**(100.0)**

**Figure 1:**
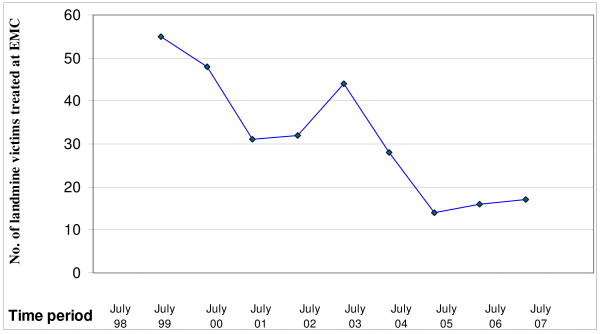
**Number of landmine injured victims admitted to EMC from July 1998 to July 2007**.

**Figure 2:**
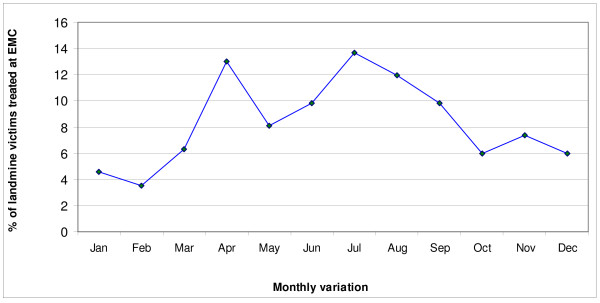
**Monthly variation of hospitalization of landmine victims to EMC**.

The number of admissions for landmine injuries was steadily decreasing between July 1998 and July 2001, followed by a transient increase between July 2002 and July 2003 (Figure 1). The highest proportion of admissions occurred during July (13.7%), followed by April (13.0%), August (11.9%), June and September (9.8% for each). In terms of seasonal variation, the highest proportion of admissions was reported in summer during June, July and August (35.4%). These findings are shown in Figure 2.

A high proportion of landmine injuries occurred from 7 am to 6 pm with the highest proportion being in the morning between 7 and 12 am (42.5%). Table 2 shows the diurnal variation of landmine injuries.

**Table 2:** Diurnal variation of landmine injury

Time of landmine injury	Male		Female		Total	
	
	**No**.	(%)	**No**.	(%)	**No**.	(%)
1 am-6 am	29	(10.7)	2	(14.3)	31	(10.9)
7 am-12 am	115	(42.4)	6	(42.9)	121	(42.5)
1 pm - 6 pm	102	(37.6)	4	(28.6)	106	(37.1)
7 pm - 12 pm	25	(9.2)	2	(14.3)	27	(9.5)
**Total**	**271**	**(100.0)**	**14**	**(100.0)**	**285**	**(100.0)**

**Figure 3:**
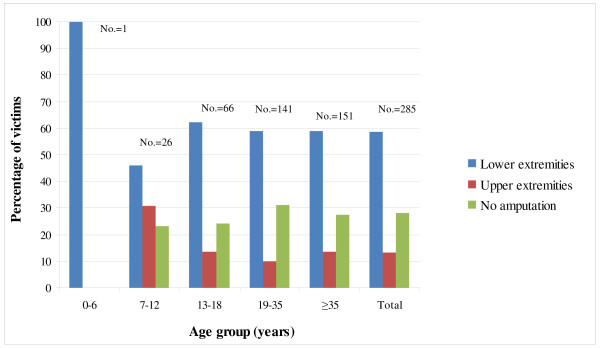
**Distribution of types of landmine injuries according to age of the victims**.

Landmine injuries caused limb amputation in 71.9% of cases; lower limb amputations and upper limb amputations constituted 58.6% and 13.3% of total cases, respectively. Hand amputation was the most common type of upper limb amputations and below-knee amputation was the most common type of lower limb amputations constituting 8.8% and 30.5% of total cases, respectively. The remaining 28.1% of cases sustained injury to different parts of the body but without limb amputation involving mainly head and face and thorax (12.3% and 5.3% of total cases, respectively). Table 3 shows details of type of injury among landmine victims. Out of the 285 patients, 19 sustained eye injuries and consequently 4 patients developed blindness. Upper limb amputations were more common among the 7-12 years age group, constituting 30.8%, than other age groups. Lower limb amputations were more common among all age groups constituting 62.1% of cases in those 13-18 years age after exclusion of one case reported in those less than 7 years. Figure 3 shows the type of injury according to the age groups of victims.

**Table 3:** Details of type of injury among landmine victims

Type of injury	**No**.	(%)	Remark
**Upper limb amputation**			
Below elbow	11	(3.9)	
Hand	25	(8.8)	
Fingers	2	(0.7)	
**Total**	**38**	**(13.3)**	

**Lower limb amputation**			
Above knee	16	(5.6)	
Below knee	87	(30.5)	
Foot	57	(20.0)	
Toes	7	(2.5)	
**Total**	**167**	**(58.6)**	

No amputation*			
Head and face	35	(12.3)	
Thorax	15	(5.3)	
Abdomen	10	(3.5)	
Back	7	(2.5)	
Upper extremities	10	(3.5)	Fracture: 2
Lower extremities	14	(4.9)	Fracture: 2
**Total**	**80**	**(28.1)**	

**Grand total**	**285**	**(100.0)**	

* Victims sustaining main injury to more than one part of body have been counted more than once

Most of the landmine accidents occurred in mountainous areas along the border areas with Iran and Turkey (61.8%) followed by injuries occurred at the areas along the former green zone between the self-administered Kurdistan region and the southern part of Iraq between 1991 and 2003 (16.8%); 14.74 occurred in the mountainous areas other than the borders and 6.7% in both urban and rural areas of Erbil city.

Out of the total hospitalized victims of landmine injuries 6 patients died with an overall mortality of 2.1%. All of these patients sustained severe injuries including bilateral above-knee amputations with injury to chest and/or abdomen. All deaths occurred within 24 hours of admission. The duration of hospital stay for the first admission of patients ranged between 1 and 121 days with a median of 13 days. The median travelling time from site of incident to the EMC was 5 hours with a range of 30 minutes to 48 hours. Nearly 38% of victims needed 6 hours or more to reach the EMC and nearly 48% of them received first aid treatment at peripheral health care facilities. Such treatment was mainly provided at Soran first aid post, two driving hours north to Erbil city.

## Discussion

This study demonstrates that civilians, males and adolescents and young adults constituted the majority of victims hospitalized for landmines injuries in Erbil governorate. It also revealed that a high proportion of these victims sustained limb amputations with lower limbs more commonly involved. However, upper limb amputations were also relatively common particularly among children. Injuries without limb amputations were also common involving mainly head and face. The hospital mortality rate was relatively low and involved mainly those with severe injuries. The number of hospitalized victims witnessed a steadily decreasing trend between July 1998 and July 2001, followed by prominent increase between July 2002 and July 2003. The highest proportion of admissions was reported in the summer months and the incidents have occurred mainly in areas located along the borders with Iran and Turkey.

This study adds to limited existing knowledge about landmine injuries in Iraq. It provides an insight to the problem through defining its magnitude, the number of victims hospitalized for landmine injuries during the study period and identifying a number of potential risk factors for injury. However, the study has a number of limitations. Data on the circumstances of injury, i.e. type of activity that resulted in the incident, occupation and level of education of victims and type of landmines were not available to be included in the study.

Another major limitation is that only hospitalized victims that had access to EMC were captured, while those with minor injuries, fatal injuries and those had no means or resources to be treated at EMC would not have been captured. Ascertainment that the injury was indeed caused by landmine and not UXO is another limitation of this study. The study was limited to Erbil governorate, whereas landmines are also abundant in the other two governorates of Iraqi Kurdistan; Duhok and Sulaimaniya.

Studies from similar and different contexts agree with the findings that the majority of victims of landmines were males and adolescents or young adults [7,10]. However, a higher percentage of injured children was reported by other studies ranging between 25% to 46%, which is probably attributed to including UXO injuries that are more common among children [1,8].

The high percentage of victims sustaining amputation to the lower limbs agrees with another study from Iran, which reported that 54.4% of landmine victims sustained amputation to the lower extremities [14]. This study showed a considerably higher percentage of victims sustaining amputation to the upper extremities as well as injury to head, face and eyes than a previous study from Iraq [10]. The low mortality rate at EMC corresponds to that in hospital setting revealed by studies from other countries [14]. This low rate does not necessarily reflect the actual mortality and severity of landmine injuries as injured patients who managed to reach the hospital may have sustained mild injuries and consequently the prehospital mortality, which is expected to be high, was not included in the study. Two other studies from Iran and Iraqi Kurdistan reported a high pre-hospital mortality rate among landmine casualties of 40% and 36.4%, respectively [14,15].

The median travel time of 5 hours revealed by this study is one of the most extreme reported travel times. While this study reports that around 38% of victims needed 6 hours or more of travel time to reach EMC, other studies from different contexts have reported that only 25% of victims need 6 hours or more to reach a hospital [1].

The decrease in number of hospitalized victims of landmine injuries between July 1998 and July 2002 might be attributed to landmine clearance activities and implementation of mine risk education programs. These activities started in Iraqi Kurdistan by a number of non-governmental organizations in mid 1990 s and boosted through the United Nations Mine Action Program during the period from 1999 to 2003. The increase in admission between July 2002 and July 2003 is mainly related to 2003 war and its aftermaths in terms of population movement and return of displaced people to areas used to be dispute areas or military bases. However, the continuous occurrence of hospitalization for landmine injured victims between 2004 and July 2007 suggests that landmines continue to represent an important health and humanitarian concern in Iraqi Kurdsitan.

The highest proportion of hospitalization of landmine injured victims noticed during April, July and August and to less extent in June and September was probably attributed to the social and economic activities undertaken in fields, hills and mountains during these months like outing, food collection, animal husbandry and agricultural work. Similarly, the occurrence of high proportion of hospitalization between 6 am and 7 pm could also be attributed to such activities, a finding which further corroborates the demonstration that most of the landmine affected individuals being productive members of the society, which agrees with the findings of other studies [7,9,10].

The effect of landmines goes beyond injury and death of victims to permanent disability of victims; this study revealed that around 72% of victims had suffered a limb amputation and 19 victims had suffered eye injury of which 4 developed blindness. Such permanent disability has profound social and economic adverse effects on the victims, their families, their communities and the local health facilities. Sustaining injuries to upper limbs and other parts including injuries to face suggests that these victims had directly dealt with or handled landmines, which could be either through playing with or trying to dismantle landmines. This finding is further corroborated by the demonstration of a high proportion of upper limp amputations among children of 7-12 years of age in comparison with other age groups. These types of injuries were observed throughout the study period indicating the importance of this issue from socioeconomic and public health point of view.

Areas along the borders and other mountainous areas, where previous military bases are abundant, are important sites for and occurrence of accidents. Areas along the former green zone between Iraqi Kurdistan and the southern part of Iraq have witnessed a considerably high number of landmine accidents since the 2003 War.

In spite of the finding that only 48% of casualties have received first aid treatment at Soran first aid post and that Soran is around 2 hours drive from the EMC, the importance of having such centre functioning properly and professionally should be emphasized. However, the travelling time from different sites of landmine accidents to Soran first aid post is around 3-4 hours. Having first aid posts functioning at different districts and sub-districts may significantly contribute to better management of landmine victims and decreasing pre-hospital mortality rate as reported by other studies [15].

## Conclusions

Civilian male adolescents and young adults constituted the majority of hospitalized landmine victims in Erbil governorate. While a high proportion of victims sustained lower limb amputations, upper limb amputations particularly among children and injury to head and face were relatively common which might be attributed to handling explosives. Therefore, there is a need to examine the reasons behind handling explosives particularly among children, whether for fun, to collect metal for cash or for other reasons, and address the risk factor accordingly. Moreover, mine risk education should target children, particularly boys, which should focus on avoiding explosives. The capacities of first aid facilities in remote areas need to be strengthened. Further research is needed to assess prehospital mortality of landmine injuries, the circumstances leading to injury and occupation and level of education of victims and whether the victims had had mine risk education.

## Abbreviations

EMC: Emergency Management Center; UXO: Unexploded ordinance

## Competing interests

The authors declare that they have no competing interests.

## Authors' contributions

The three authors participated in designing the study. SNP and THI carried out the data collection. AH TS and SNP carried out the data analysis. SNP and THI drafted the first version of the paper. AHTS extensively reviewed the first draft and made comprehensive changes. All three authors reviewed the final draft and approved it.
